# Potassium Currents Activated by Depolarization in Odontoblasts

**DOI:** 10.3389/fphys.2017.01078

**Published:** 2017-12-19

**Authors:** Yuki Kojima, Maki Kimura, Asuka Higashikawa, Kyosuke Kono, Masayuki Ando, Masakzu Tazaki, Yoshiyuki Shibukawa

**Affiliations:** Department of Physiology, Tokyo Dental College, Tokyo, Japan

**Keywords:** dentinogenesis, K^+^ channels, Kv channels, odontoblast, outward currents, patch-clamp recordings

## Abstract

Increased intracellular free Ca^2+^ concentrations elicit plasma membrane depolarization, which leads to the activation of K^+^ currents. However, the precise properties of K^+^ currents activated by depolarization in odontoblasts remain to be elucidated. The present study identified biophysical and pharmacological characteristics of time-dependent and voltage-activated K^+^ currents in freshly dissociated rat odontoblasts using patch-clamp recordings in a whole-cell configuration. Using a holding potential of −70 mV, outwardly rectifying time- and voltage-dependent currents were activated by depolarizing voltage. To record pure K^+^ conductance, we substituted Cl^−^ in both the extracellular and intracellular solutions with gluconate^−^. Under these conditions, observation of K^+^ concentration changes in the extracellular solution showed that reversal potentials of tail currents shifted according to the K^+^ equilibrium potential. The activation kinetics of outward K^+^ currents were relatively slow and depended on the membrane potential. Kinetics of steady-state inactivation were fitted by a Boltzmann function. The half-maximal inactivation potential was −38 mV. Tetraethylammonium chloride, 4-aminopyridine, and α-dendrotoxin inhibited outward currents in odontoblasts in a concentration-dependent manner, suggesting that rat odontoblasts express the α-subunit of the time- and voltage-dependent K^+^ channel (Kv) subtypes Kv1.1, 1.2, and/or 1.6. We further examined the effects of Kv activity on mineralization by alizarin red and von Kossa staining. Continuous application of tetraethylammonium chloride to human odontoblasts grown in a mineralization medium over a 21-day period exhibited a dose-dependent decrease in mineralization efficiency compared to cells without tetraethylammonium chloride. This suggests that odontoblasts functionally express voltage-dependent K^+^ channels that play important roles in dentin formation.

## Introduction

Odontoblasts are located at the inner surface of dentin, which forms the most outer surface of dental pulp. These cells secrete dentin matrix proteins and are responsible for mineralization during physiological developmental/aging processes and as a result of stimuli being applied to the outer surface of dentin (such as tertiary dentin formation, specifically reactionary dentin formation). In addition to dentin formation (dentinogenesis), recent convergent evidence indicates that odontoblasts also act as sensory receptor cells and play important roles in the generation of dentinal pain (Magloire et al., 2010; Shibukawa et al., 2015; Nishiyama et al., 2016). As sensory receptor cells, odontoblasts are capable of detecting cellular deformation induced by mechanical stimuli (i.e., the change of hydrodynamic force inside dentinal tubules) via various mechanosensitive ionic channels, including the transient receptor potential (TRP) channel family and Piezo channels (Okumura et al., 2005; Magloire et al., 2010; Tsumura et al., 2012, 2013; Sato et al., 2013, 2015; Shibukawa et al., 2015; Kimura et al., 2016; Nishiyama et al., 2016). In addition, odontoblasts also express various ionotropic and/or metabotropic receptors for extracellular signaling molecules, including nucleotides (ATP/ADP), glutamate, muscarine, cannabinoids, and bradykinin (Shibukawa and Suzuki, 2003; Korkmaz et al., 2006; Ichikawa et al., 2012; Tsumura et al., 2012; Sato et al., 2015; Shibukawa et al., 2015; Nishiyama et al., 2016; Shiozaki et al., 2017). Activation of these mechanosensitive ionic channels, as well as ionotropic and metabotropic receptors, increases intracellular free Ca^2+^ concentrations, leading to plasma membrane depolarization. Membrane depolarization in odontoblasts is known to activate K^+^ conductance; however, the detailed properties of this process remains to be clarified (Kojima et al., 2015).

Significant outward current was generated by time- and voltage-dependent K^+^ channels not only in the most excitable, but also in the non-excitable cells, and also regulate membrane potential (Coetzee et al., 1999; c.f. Hille, 2001). The α-subunit family of the time- and voltage-dependent K^+^ channels (Kv) is the largest gene family of K^+^ channels (Gutman et al., 2005), being encoded by 40 different genes and categorized into 12 sub-families, Kv1 through Kv12. Kv constitutes a six-transmembrane group of K^+^-selective channels consisting of six membrane-spanning domain subunits, including the S4 segment, which acts as a voltage sensor on some of these channels. Individual Kv channel family members can be identified based on the different properties with respect to their time and voltage dependency for activation and inactivation kinetics, their pharmacological characteristics, and their molecular marker sensitivity. The K^+^ current mediated by Kv has been classified into five groups according to their electrophysiological properties: “A-type,” “delayed rectifier type,” “modifier/silencer type” and “inwardly rectifying type” currents (Gutman et al., 2005). For example, Shaker-related Kv channels (Kv1.1, Kv1.3, Kv1.2, Kv1.5, Kv1.6, Kv1.7, and Kv1.8) exhibit “delayed rectifier” properties, when present as a homotetramer.

To identify the biophysical properties governing the currents generated by time- and voltage-dependent K^+^ channels in freshly isolated rat odontoblasts, we studied the kinetics of activation and inactivation and determined the steady-state characteristics of these K^+^ currents using whole-cell patch-clamp recordings. Additional studies demonstrated ionic selectivity upon changes in [K^+^]_o_ and the pharmacological profile of these K^+^ currents. We also examined the effects of blocking time- and voltage-dependent K^+^ channels on human odontoblast-driven mineralization.

## Materials and methods

### Ethical approval

The animal experimental protocol was approved by the Ethics Committee of our institute (No. 270301, No. 280301, and No. 290303). This study was performed according to the Guiding Principles for the Care and Use of Animals in the field of physiological sciences, as approved by the Council of the Physiological Society of Japan and the American Physiological Society. All animal experiments followed the guidelines established by the U.S. National Institutes of Health regarding the care and use of animals for experimental procedures, as well as the UK Animals (Scientific Procedures) Act, 1986.

### Dental pulp slice preparation

We obtained dental pulp slice preparations from neonatal Wistar rats (6- to 7-days-old) using a previously described method (Shibukawa and Suzuki, 2003; Tsumura et al., 2010, 2012, 2013; Shibukawa et al., 2015; Kimura et al., 2016). Animals were anesthetized using isoflurane (3%) and pentobarbital sodium (25 mg/kg), and the mandible was dissected. The mandible was embedded in an alginate impression material, and 500-μm thick slices were obtained by transversely sectioning tissue through the incisors using a standard vibrating tissue slicer (Dosaka EM, Kyoto, Japan). The mandible was sectioned to the level where the dentin and enamel were directly visible between the bone tissue and the dental pulp. The surrounding impression material, bone tissue, enamel, and dentin were carefully removed from these mandible sections under a stereoscopic microscope, and the residual dental pulp slice was used for further experiments. We selected mandible sections with a thin dentin layer to avoid cellular damage to the odontoblasts, but with enamel and dentin that could clearly be distinguished under a microscope. Pulp slices were treated with a standard Krebs solution containing 0.03% trypsin and 0.17% collagenase at 37°C for 30 min. For patch-clamp recording, enzymatically treated dental pulp slices were plated onto a culture dish, immersed in alpha-minimum essential medium (Thermo Fisher Scientific, Waltham, MA, USA), supplemented with 10% fetal bovine serum and 5% horse serum, and maintained at 37°C in a 5% CO_2_ incubator. Primary cultured dental pulp slices were used for patch-clamp recording within 24 h of isolation.

### Culture of human odontoblasts

An immortalized cell line derived from dental pulp of the human molar with odontoblastic characteristics (HOB cells; Kitagawa et al., 2007; Ichikawa et al., 2012; Kimura et al., 2016) was cultured in basal media containing alpha-minimum essential medium, 10% fetal bovine serum, and 1% penicillin/streptomycin (Thermo Fisher Scientific).

### Whole-cell patch-clamp recording

Whole-cell recordings (Hamill et al., 1981) of odontoblasts in the primary cultured dental pulp slices were conducted through voltage-clamp recording under patch-clamp technique using a conventional method. Cells located in the rim of cultured dental pulp slices positively expressed odontoblast marker proteins of dentin matrix protein-1, dentin sialoprotein, and nestin (Tsumura et al., 2012), indicating that these cells were odontoblasts. Patch pipettes, with a resistance of 3 to 8 MΩ, were made from glass capillary tubes (DMZ-Universal Puller; Zeitz-Instruments, Martinsried, Germany) and filled with an intracellular solution. When the patch pipette was attached onto the plasma membrane, we immediately measured the resistance (seal resistance) between the pipette and membrane. The mean value of initial seal resistance was 2.3 ± 0.7 GΩ (“gigaseal”; *N* = 51). The membrane resistance of the cells during whole-cell recording was calculated from the current amplitude evoked by a 10 mV depolarizing voltage step from a Vh of –70 mV. The mean value of membrane resistance was 988.1 ± 112.3 MΩ (*N* = 51). We measured whole-cell currents with an amplifier for patch-clamp recordings (L/M-EPC-7 plus; HEKA Elektronik, Lambrecht, Germany). After digitization of the analog signals at 10 kHz (Digidata 1440A; Molecular Devices, Sunnyvale, CA), current traces were monitored and stored using pCLAMP (Molecular Devices). Data were analyzed with pCLAMP and the technical graphics/analysis program, ORIGIN, on an offline computer (OriginLab Corporation, Northampton, MA, USA). All experiments were performed at 25°C. We calculated the membrane capacitance of odontoblasts using the capacitative transient current induced by depolarizing steps (10 mV) starting from a holding potential (Vh) of 0 mV. Small differences in odontoblast size were accounted for by normalizing the measured capacitance and expressing current amplitudes in terms of current densities (pA/pF).

### Mineralization assay

Cultured HOB cells were grown to full confluency in basal media and then grown in mineralization media, containing 10 mM β-glycerophosphate and 100 μg/mL ascorbic acid (final concentration) in basal media, at 37°C with 5% CO_2_. To examine the inhibitory effects of voltage-dependent K^+^ channels on mineralization by odontoblasts, tetraethylammonium chloride (TEA; 2 or 4 mM, *N* = 6, respectively) was applied to the mineralization medium over a 21 day period. We exchanged the medium once every 3 days. To detect calcium deposits, cells were subjected to alizarin Red and von Kossa staining (Suzuki et al., 2014; Chen et al., 2016; Kimura et al., 2016).

### Solutions and reagents

Krebs solution, containing 136 mM NaCl, 5 mM KCl, 2.5 mM CaCl_2_, 0.5 mM MgCl_2_, 10 mM HEPES, 10 mM glucose, and 12 mM NaHCO_3_ (pH 7.4 by Tris) was used as the standard extracellular solution (ECS) and Cl^−^-rich ECS for patch-clamp recording. The Cl^−^-rich intracellular solution (ICS) contained 140 mM KCl, 10 mM NaCl, and 10 mM HEPES (pH 7.2 by Tris). For patch-clamp recording under physiological conditions, we used solutions of Cl^−^-rich ECS and Cl^−^-rich ICS. To record pure K^+^-conductance, we substituted NaCl in the Cl^−^-rich ECS and KCl in the Cl^−^-rich ICS with Na-gluconate and K-gluconate, respectively (gluc-rich ECS/ICS). TEA and 4-aminopyridine (4-AP) were obtained from Wako Pure Chemicals (Osaka, Japan). α-Dendrotoxin (αDTX) was obtained from Alomone Laboratories (Jerusalem, Israel). We prepared stock solutions of these reagents in distilled water. The stock solutions were then diluted with ECS to the appropriate concentration immediately before the experiments. We purchased all other reagents from Sigma Chemical Co. (St. Louis, MO, USA).

### Statistics

We expressed the results as mean ± standard deviation (SD) for an N number of observations. We represented the number of tested cells as N. The Wilcoxon signed-rank test or Steel–Dwass multiple comparisons were used to evaluate non-parametric statistical significance. Values of *P* < 0.05 were considered significant.

## Results

### Passive membrane properties of acutely isolated odontoblasts

We measured the resting membrane potential (*Rm*) immediately after establishing the whole-cell recording configuration using conventional method. The averaged *Rm* value was −56.2 ± 5.3 mV (*N* = 19) in Cl^−^-rich ECS (with extracellular 5 mM KCl) and Cl^−^-rich ICS. These isolated odontoblasts had a membrane capacitance of 13.1 ± 2.5 pF (*N* = 19) under physiological conditions.

### Outward currents in odontoblasts

**Figure 1:**
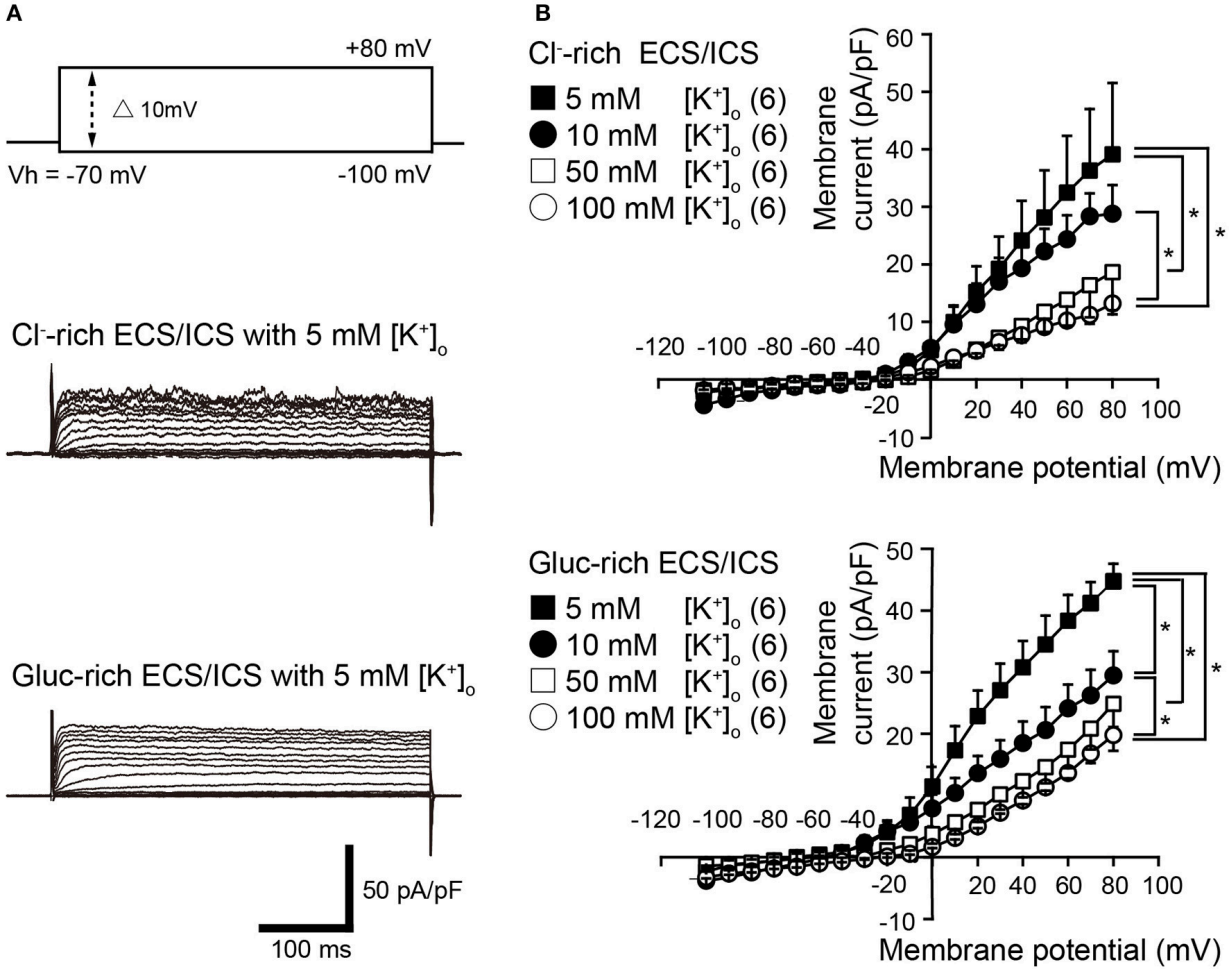
Outwardly rectifying currents in odontoblasts. **(A)** Representative current traces with extracellular 5 mM [K^+^]_o_ recorded in Cl^−^-rich extracellular and intracellular solutions (Cl^−^-rich ECS and ICS) (middle traces), as well as in gluconate-rich ECS and ICS (Gluc-rich ECS and ICS) (lower traces) are shown. Currents were evoked by 400 ms voltage-steps from −100 to +80 mV in 10 mV increments from a Vh of −70 mV (upper traces). **(B)** Current-voltage (I-V) relationships for the current recorded in Cl^−^-rich ECS and ICS (upper), and gluc-rich ECS and ICS (lower) with 5 mM [K^+^]_o_ (filled squares), 10 mM [K^+^]_o_ (filled circles), 50 mM [K^+^]_o_ (open squares), and 100 mM [K^+^]_o_ (open circles). Each point indicates the current density for the mean ± SD from each of the 6 cell. Statistically significant differences between points at a membrane potential of +80 mV (shown by solid lines) are indicated by asterisks, ^*^P < 0.05.

Voltage steps (400 ms in duration) ranging from −100 to +80 mV in 10 mV increments, from a holding potential (Vh) of −70 mV (upper traces in Figure 1A), elicited time-dependent outward currents in both the physiological Cl^−^-rich ECS/ICS (middle traces in Figure 1A) and gluc-rich ECS/ICS with an extracellular K^+^ concentration ([K^+^]_o_) of 5 mM (lower traces in Figure 1A). We obtained current-voltage (I–V) relationships (Figure 1B) by plotting the amplitude of the steady-state component of these currents against the membrane potential in the presence of four different [K^+^]_o_ ranging from 5 to 100 mM. The outward current activation threshold showed a 10 mV negative shift for the current recorded under gluc-rich conditions, compared to those measured in Cl^−^-rich ECS/ICS (Figure 1B). The I-V relationships for the currents recorded in both the Cl^−^-rich ECS/ICS and gluc-rich ECS/ICS, with various [K^+^]_o_ (5–100 mM), showed outward rectification (Figure 1B). The amplitudes of outward currents in both the Cl^−^-rich and gluc-rich conditions decreased as [K^+^]_o_ increased, suggesting the currents were carried by K^+^. Each activation threshold value for the outward currents, in both the physiological and gluc-rich ECS/ICS supplemented with 100 mM [K^+^]_o_, also showed a shift toward depolarizing membrane potentials compared to the value recorded with 5 mM [K^+^]_o_.

### K^+^ selectivity of the outward currents in odontoblasts

**Figure 2:**
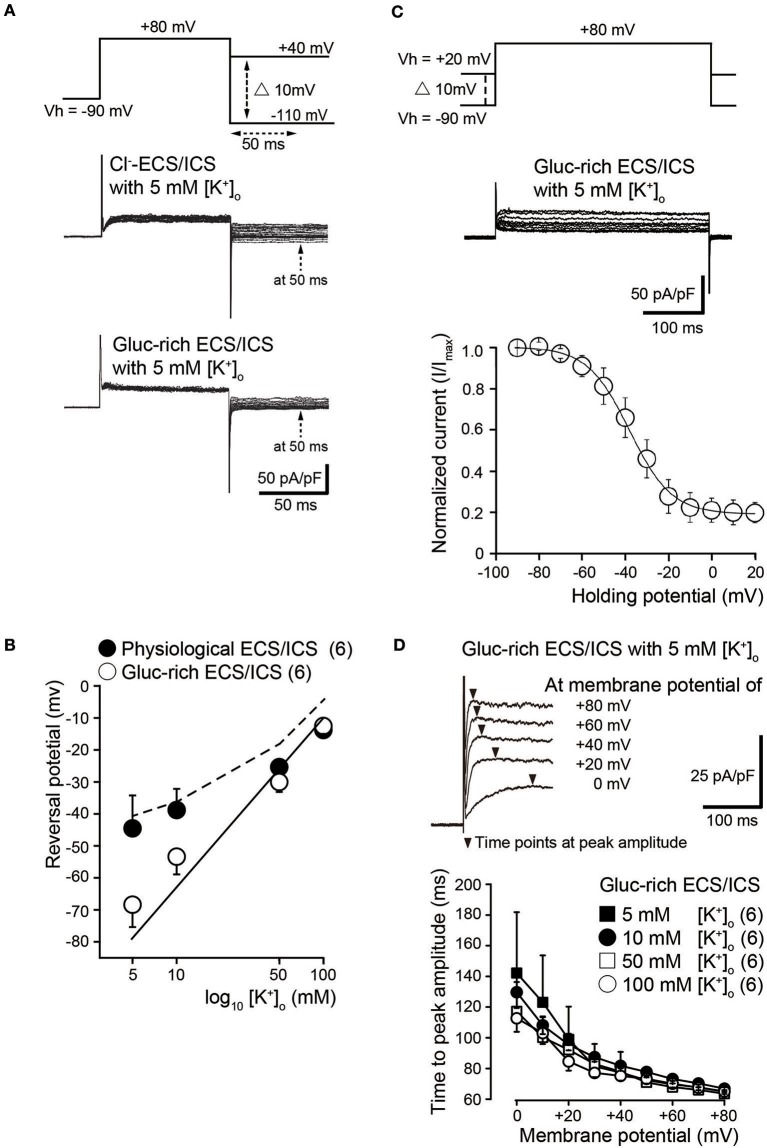
Biophysical properties of outward currents in odontoblasts. **(A)** Representative current traces recorded under conditions of Cl^−^-rich ECS and ICS (middle traces) as well as gluc-rich ECS and ICS (lower traces) are shown. Currents were elicited by voltage steps from a Vh of −70 mV to +80 mV before hyperpolarizing in 10 mV increments ranging from −110 to +40 mV (upper traces). To analyze the values of reversal potential, we measured the amplitudes at 50 ms (dashed arrows in both the middle and lower) after initiating hyperpolarized voltage pulsing (dashed double headed arrow in upper) under both conditions. **(B)** Semilogarithmic plots of reversal potential against various [K^+^]_o_ (5–100 mM) under Cl^−^-rich physiological (filled circles) and gluc-rich (open circles) conditions are shown. The reversal potentials under gluc-rich conditions were closely fitted to expected values, which were estimated for pure K^+^ conductance described by the Nernst equation (solid line). The dashed line shows the reversal potentials estimated by the Goldman-Hodgkin-Katz equation with a Cl^−^ permeability of 0.19 ± 0.16 (K^+^ permeability was set to 1.0). Each point indicates the reversal potentials for the mean ± SD from each 6 cell. **(C)** Representative current traces for steady-state inactivation recorded under gluc-rich ECS and ICS conditions (middle traces) are shown. Currents were evoked by incrementally stepping to a membrane potential of +80 mV from various starting Vh values, which varied from −90 to +20 mV (upper traces). In each recording, Vh was maintained for at least 30 s before applying the depolarizing step. Relationships between normalized peak current amplitudes (I/I_max_; see text) under gluc-rich conditions and various applied holding potentials were well fitted to the Boltzman function (*Equation 1*). **(D)** Representative current traces in the presence of 5 mM [K^+^]_o_ recorded under glu-rich ECS and ICS conditions evoked by voltage-steps from 0 to +80 mV in 20 mV increments from a Vh of −70 mV are shown. To analyze activation kinetics, time to peak current amplitudes were measured (time from initiation of voltage pulse to peak current amplitudes; filled triangles) in the presence of 5 mM [K^+^]_o_ (filled squares), 10 mM [K^+^]_o_ (filled circles), 50 mM [K^+^]_o_ (open squares), and 100 mM [K^+^]_o_ (open circles), and subsequently plotted against membrane potentials. Each point indicates the values for the mean ± SD from each 6 cell.

To investigate ion selectivity of the outward currents, we analyzed tail currents and measured their reversal potentials at four different [K^+^]_o_: 5, 10, 50, and 100 mM. In each experiment, tail current families were elicited by voltage steps (upper traces) from Vh of −70 mV to +80 mV before hyperpolarizing in 10 mV increments from −110 to +40 mV under both Cl^−^-rich (middle traces) and gluc-rich conditions (lower traces) (Figure 2A). We measured tail current amplitudes 50 ms after the start of the hyperpolarizing pulse (dashed arrows in Figure 2A). We plotted the amplitudes as a function of the applied membrane potential and subsequently obtained the reversal potentials. The mean reversal potential values under physiological conditions were −44.5 ± 10.2 mV in 5 mM [K^+^]_o_, −38.8 ± 6.6 mV in 10 mM [K^+^]_o_, −25.3 ± 2.1 mV in 50 mM [K^+^]_o_, and −13.8 ± 2.4 mV in 100 mM [K^+^]_o_ (filled circles in Figure 2B; *N* = 6). The mean reversal values in the gluc-ECS/ICS were −68.3 ± 7.0 mV in 5 mM [K^+^]_o_, −53.3 ± 5.6 mV in 10 mM [K^+^]_o_, −30.0 ± 3.2 mV in 50 mM [K^+^]_o_, and −12.5 ± 2.5 mV in 100 mM [K^+^]_o_ (open circles in Figure 2B; *N* = 6).

Semilogarithmic plots of the reversal potentials against [K^+^]_o_ (5–100 mM) demonstrated that the mean reversal potential values in the gluc-rich ECS/ICS were similar to those expected for a current showing K^+^-selectivity, as estimated by the Nernst equation at 25°C (Figure 2B, solid line) by assuming an intracellular K^+^ concentration of 140 mM (see Materials and Methods). We also observed deviations of the reversal potentials recorded under physiological conditions from those estimated by the Nernst equation for pure K^+^ conductance. The Cl^−^ permeability was estimated according to the Goldman-Hodgkin-Katz equation from recorded reversal potentials under physiological conditions, where the extracellular Cl^−^ concentration ([Cl^−^]_o_) was 147 mM, the intracellular Cl^−^ concentration was 150 mM, and the [K^+^]_i_ was 140 mM with various [K^+^]_o_ concentrations. Under these conditions, we estimated a Cl^−^ permeability of 0.15 in 5 mM [K^+^]_o_, 0.17 in 10 mM [K^+^]_o_, 0.03 in 50 mM [K^+^]_o_, and 0.42 in 100 mM [K^+^]_o_. The overall mean Cl^−^ permeability value was 0.19 ± 0.16 (K^+^ permeability was set to 1.0), with the estimated reversal potential values being −41.4 mV ([K]_o_ = 5 mM), −37.8 mV ([K]_o_ = 10 mM), −19.5 mV ([K]_o_ = 50 mM), and −6.9 mV ([K]_o_ = 100 mM) (dashed line in Figure 2B).

### Steady-state inactivation properties of K^+^ currents

We examined the voltage dependence of the steady-state inactivation kinetics of outward K^+^ currents in gluc-rich ECS/ICS. We obtained a family of currents (middle traces in Figure 2C) by a voltage step to a membrane potential of +80 mV from various starting Vh values, which varied from −90 to +20 mV (upper traces in Figure 2C). In each recording, the Vh was maintained for 30 s before applying the depolarizing step. As expected, the outward current amplitude decreased as the holding potential became more positive. Current amplitudes for a given holding potential (I) were normalized to the amplitude at the holding potential of −90 mV (I_max_). The normalized values (I/I_max_) were subsequently plotted against the selected Vh values (Figure 2C). The voltage dependence of the steady-state inactivation of outward K^+^ currents was obtained by fitting the data using a Boltzmann function:

(1) $$\text{I}/{\text{I}}_{\text{max}}=1/\{1+\text{exp}[\left({\text{V}}_{\text{h}}-{\text{V}}_{0.5}\right)/\text{k}]\}$$

where V_h_ is the holding potential, V_0.5_ is the membrane potential at which the channels are inactivated by 50%, and *k* is the slope factor. The best fits of V_0.5_ were −38.0 ± 1.0 mV in 5 mM [K^+^]_o_ (*N* = 6).

### Activation kinetics of voltage-dependent K^+^ currents in odontoblasts

To analyze activation kinetics, we measured the time to peak current amplitudes at membrane potentials from 0 to +80 mV in gluc-rich ECS/ICS with the addition of a range of [K^+^]_o_ (5–100 mM). The time to peak current amplitude values significantly decreased as the membrane potential became more positive. Each time to peak current amplitude for activation with different [K^+^]_o_ vs. membrane potentials showed strong dependence on the membrane potential (Figure 2D). There were no significant differences in the values using the different [K^+^]_o_ (5–100 mM).

### Pharmacological properties of voltage-dependent K^+^ currents expressed in odontoblasts

**Figure 3:**
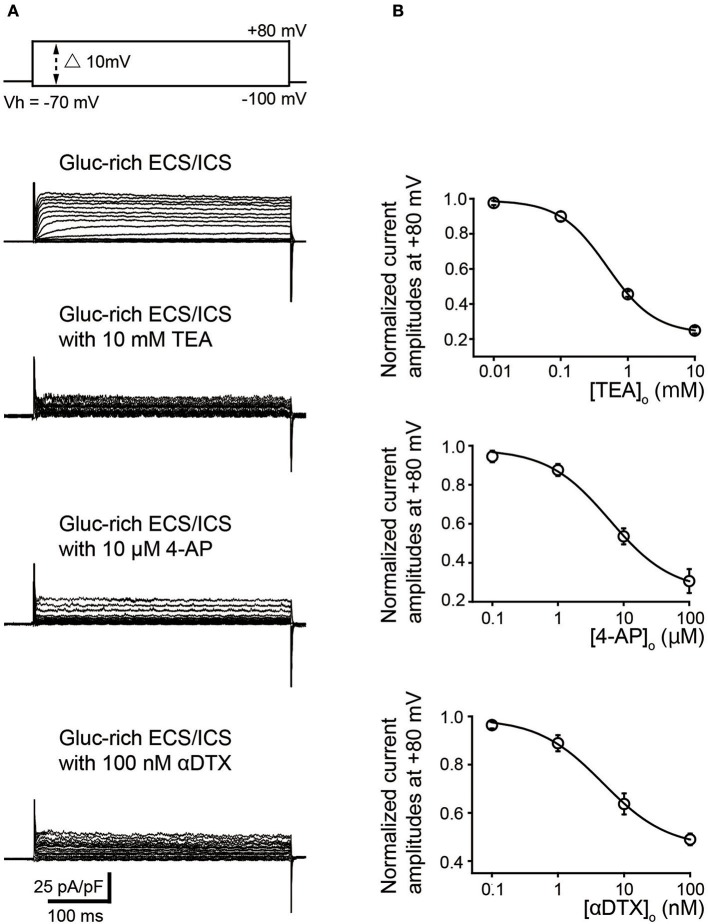
Pharmacological properties of outward currents in odontoblasts. **(A)** Representative current traces recorded under gluc-rich ECS and ICS conditions without (second upper traces) and with 10 mM TEA (third upper traces), 10 μM 4-AP (forth upper traces), and 100 nM αDTX (bottom traces) are shown. Currents were evoked by 400 ms voltage-steps from −100 to +80 mV in 10 mV increments from a Vh of −70 mV (upper traces). **(B)** Dose-response relationships between current amplitudes normalized to those without antagonist and those with various concentration of extracellular antagonist: TEA (upper), 4-AP (middle), and αDTX (lower). Each data point was best fitted using *Equation 2*, and each IC_50_ value was evaluated. Each point indicates the mean values ± SD from each 6 cell.

We further examined the effect of pharmacological K^+^ channel blockers to identify the α-subunit of the time- and voltage-dependent K^+^ channels (Kv). Outward K^+^ currents (Figure 3) in the gluc-rich ECS/ICS supplemented with 5 mM [K^+^]_o_ were activated by depolarizing voltage steps (400 ms in duration) ranging from −100 to +80 mV, in 10 mV increments, from a Vh of −70 mV (most upper traces) before, during, and after application of Kv inhibitors. TEA and 4-AP, which are both non-selective Kv blockers, attenuated outward current amplitudes at a membrane potential of +80 mV in a dose dependent manner. Peak current amplitudes at +80 mV were significantly inhibited by 10 mM TEA (75.0 ± 1.8%) and 100 μM 4-AP (69.4 ± 6.1%) (Figure 3B; *N* = 6). An inhibitor of Kv1.1, Kv1.2, and Kv1.6, αDTX (100 nM), significantly suppressed the peak current amplitudes at a membrane potential at +80 mV to 51.0 ± 2.4% of the peak current amplitude (Figure 3B; *N* = 6). The pattern of these results suggested that odontoblasts express K^+^ currents via Kv1.1, Kv1.2, and/or Kv1.6. The effects of antagonists were evaluated by fitting the data according to the function:

(2) $$\begin{array}{l} \text{I}/{\text{I}}_{\text{cont}}=({\text{I/I}}_{\text{cont}}-{\text{I/I}}_{\text{cont min}})/[\left(1+\left({\left[\text{antagonist}\right]}_{\text{o}}/{\text{IC}}_{50}\right)\right)\\ \;+\text{I}/{\text{I}}_{\text{cont min}} \end{array}$$

where I/I_cont_ was the normalized value (of current amplitudes with various concentrations of extracellular antagonist ([antagonist]_o_) normalized to those without antagonist); I/I_cont min_ was minimal value of I/I_cont_; and IC_50_ was the half maximal (50%) inhibitory concentration of the antagonists. The IC_50_ was determined to be 0.49 ± 0.39 mM for TEA (*N* = 6; I/I_cont min_ was 0.24), 6.15 ± 1.65 μM for 4-AP (*N* = 6; I/I_cont min_ was 0.30), and 4.77 ± 0.91 nM for αDTX (*N* = 6; I/I_cont min_ was 0.45). In addition, reversal potentials recorded by 400 ms depolarizing voltage steps in the gluc-rich ECS/ICS with 5 mM [K^+^]_o_ showed a shift toward depolarizing membrane potentials to −21.8 ± 8.1 mV by 10 mM TEA, −28.2 ± 1.6 mV by 100 μM 4-AP, and – 34 ± 3.6 mV by 100 nM αDTX (*N* = 6 each).

### TEA inhibited mineralization induced by odontoblasts

**Figure 4:**
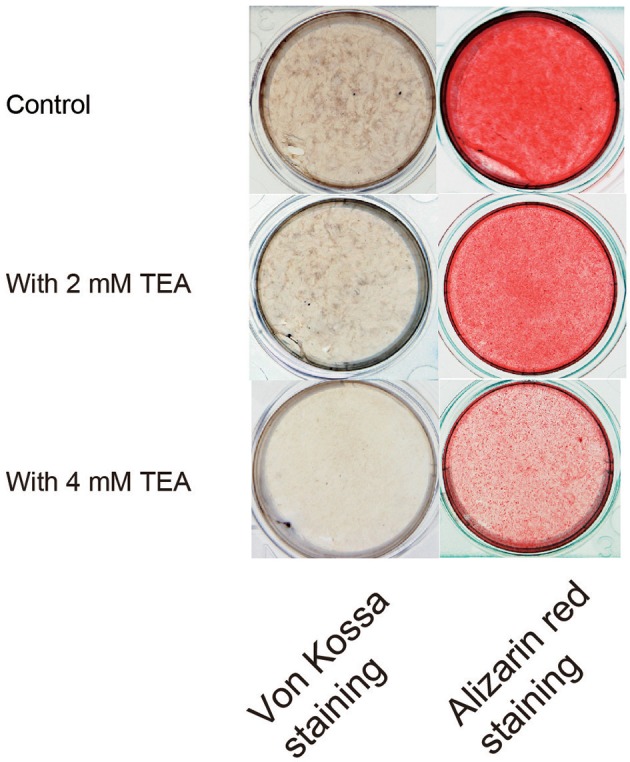
Effect of K^+^ channel activity on the mineralization. Photographs show the von Kossa (left panels) and alizarin red (right panels) staining, without (controls; upper panels) or with 2 mM (middle panels) or 4 mM (lower panels) TEA.

Using alizarin red (each right image, Figure 4) and von Kossa (each left image, Figure 4) staining, we further examined the effects of Kv activity on mineralization. The continuous application of TEA (2 mM or 4 mM) to mineralization medium containing human odontoblasts over a 21 day period showed a dose-dependent decrease in the mineralization efficiency compared to the cells without TEA (as control).

## Discussion

The present study provides detailed electrophysiological/biophysical properties of the voltage-dependent outward currents in acutely isolated rat odontoblasts. Voltage-dependent outward currents in odontoblasts showed outward rectification. Time to peak current amplitude decreased with increased membrane potential, thereby displaying voltage dependency. The values for time to peak current amplitude indicated the current to have slow-activating properties (Xu et al., 1999; Gutman et al., 2005; DiFranco et al., 2012; Zhan et al., 2012). The resting membrane potential (*Rm*) of rat odontoblast was determined to be −56 mV in this study. Although this value fell within the range reported for odontoblasts from rat, dog, and human (ranging −24 to −52 mV) (Winter et al., 1963; Lundgren and Linde, 1992; Allard et al., 2000; Ichikawa et al., 2012), the *Rm* for rat odontoblasts showed a depolarizing shift from the K^+^ equilibrium potential estimated in the presence of 5 mM [K^+^]_o_. However, for the tail current analysis, the reversal potentials in gluc-rich ECS/ICS were closely approximated to the conductance for pure K^+^, indicating that outward currents were mostly carried by K^+^ under these conditions. By contrast, the reversal potentials recorded under physiological conditions in the presence of various [K^+^]_o_ deviated from those showing pure K^+^ conductance.

The reversal potentials, estimated using the Goldman-Hodgkin-Katz equation with a Cl^−^ permeability of 0.19 (K^+^ permeability was set to 1.0), closely approximated the values herein recorded. Therefore, both the K^+^ and Cl^−^ conductance are likely included in the voltage-dependent outward currents in odontoblasts. In addition, when we applied nonspecific or specific inhibitors for Kv1.1, Kv1.2, and Kv1.6, the reversal potential showed a positive shift toward depolarizing membrane potentials. These results indicated that K^+^ conductance carried by Kv1.1, Kv1.2, and Kv1.6 plays an important role in maintaining the *Rm* in rat odontoblasts. Thus, the results suggest that Cl^−^ conductance contributed to the depolarized shift of *Rm* from the potential mediated by the pure K^+^ conductance in odontoblasts; however, the activity of Kv, including Kv1.1, Kv1.2, and Kv1.6, plays a key role in maintaining *Rm* in rat odontoblasts. These results are also in agreement with the results showing expression of not only K^+^ but also Cl^−^ channels in rat odontoblast membranes (Guo and Davidson, 1998; Shibukawa and Suzuki, 2000). In the steady-state inactivation analysis, the outward K^+^ currents showed voltage dependency with V_0.5_ of −38.0 mV. This suggests K^+^ channels in odontoblasts are capable of opening within resting membrane potentials to regulate cellular function(s).

Through a pharmacological analysis, voltage-dependent K^+^ channels in odontoblasts were shown to be sensitive to K^+^ channel inhibitors; TEA, 4-AP, and αDTX. TEA and 4-AP are non-selective Kv inhibitors, while αDTX is a selective inhibitor for Kv1.1, 1.2, and/or 1.6. Kv inhibition with αDTX indicates rat odontoblasts expresses one or all of the Kv subtypes Kv1.1, 1.2, and/or 1.6. Human odontoblasts (Ichikawa et al., 2012) express intermediate-conductance Ca^2+^-activated K^+^ channels that are sensitive to the inhibitors of Osk1 and charybdotoxin (CTX), but insensitive to iberiotoxin (ITX). These Ca^2+^-activated K^+^ channel inhibitors are also sensitive to specific types of Kv currents; CTX is a selective inhibitor of Kv1.2 and Kv1.3, while Osk1 is a potent inhibitor of Kv1.1, Kv1.2, and Kv1.3. Agitoxin, which has been used more recently as a Kv1.3 inhibitor, had no effect on depolarization-induced outward currents in human odontoblats. Although expression of Kv1.1 to 1.3 in human odontoblasts remains to be clarified, at least Kv1.1 and Kv1.2 appear to be conservatively expressed in both human and rat odontoblasts (note that Ichikawa et al., 2012 reported Agitoxin is an inhibitor for Kv1.1 to 1.3; however it has been used more recently as a Kv1.3-specific inhibitor; Anangi et al., 2012).

Although rat dental pulp fibroblasts also express voltage-dependent K^+^ channels, in contrast to odontoblasts they show transient outward currents (rapidly activating) with slow inactivation properties (Shibukawa and Suzuki, 2001). These currents are sensitive to 4-AP, but insensitive to not only TEA and blood depressing substance-I (inhibitor for Kv3), but also mast cell degranulating peptide and dendrotoxin-I (inhibitors for Kv1.1 and 1.2). It is quite interesting to examine the Kv expression differences between odontoblasts and fibroblasts; odontoblasts seem to express both Kv1.1 and 1.2, while dental pulp fibroblasts express neither. These differences might be related to the differences of cellular function. In the present study, we also observed the remaining component of outward currents in odontoblasts during the application of TEA, 4-AP, or αDTX. Therefore, our immediate subsequent interest is to clarify the detailed Kv expression patterns not only in odontoblasts, but also in dental pulp fibroblasts. In addition, we intend to investigate what influence the differences in Kv expression pattern in the respective cells might have on the cellular function.

It has been reported that voltage-dependent K^+^ channel activities were involved in mineralization processes by “osteoblasts” (Henney et al., 2009). Voltage-dependent K^+^ channel blockade in osteoblasts had a profound positive impact on mineralization process, while inhibition of voltage-dependent K^+^ channels in odontoblasts was found to inhibit the mineralization in the present study. This suggests the activation of Kv and maintenance of hyperpolarized *Rm* play important roles in dentin mineralization.

In conclusion, we showed that odontoblasts expressed slow activating voltage-dependent K^+^ currents that are carried by Kv1.1, 1.2, and/or 1.6. Previous reports have shown various Ca^2+^ influx/mobilization pathways are mediated by mechano-sensing cation channels, store-operated Ca^2+^ channels (SOCs), as well as depolarization-induced Ca^2+^ entry (Shibukawa and Suzuki, 2003; Son et al., 2009; Tsumura et al., 2010, 2013; Kojima et al., 2015; Shibukawa et al., 2015). Cellular deformation in odontoblasts, which might be elicited by dentinal fluid movement following dentin stimulation, also activates Ca^2+^ influx and leads to depolarization. Dentin stimulation is closely related to reactionary dentin formation. Depolarizing membrane potentials activates Kv in odontoblasts, leading to hyperpolarization to maintain *Rm*, with the resulting negative membrane potential shift subsequently increasing the driving force for Ca^2+^. The acceleration of Ca^2+^ influx in odontoblasts may in turn modulate enhancement of dentin formation during dentin stimulation. Kv channels in odontoblasts might play an important role in driving cellular functions, not only for the stabilization of plasma membrane potential, but also for dentinogenesis in the physiological/pathological settings.

## Author contributions

YK, MK, MT, and YS designed the study. YK, MK, AH, KK, MA, and YS acquired and analyzed the data. YK, YS, and MT interpreted the data. YK, YS, and MT drafted the manuscript. All authors read and approved the final manuscript.

### Conflict of interest statement

The authors declare that the research was conducted in the absence of any commercial or financial relationships that could be construed as a potential conflict of interest.
